# An Adaptive Unsupervised Feature Selection Algorithm Based on MDS for Tumor Gene Data Classification

**DOI:** 10.3390/s21113627

**Published:** 2021-05-23

**Authors:** Bo Jin, Chunling Fu, Yong Jin, Wei Yang, Shengbin Li, Guangyao Zhang, Zheng Wang

**Affiliations:** 1 School of Artificial Intelligence, Henan University, Kaifeng 475004, China; jinbo@henu.edu.cn (B.J.); jy@henu.edu.cn (Y.J.); shengbin-L@henu.edu.cn (S.L.); zgy20200210@henu.edu.cn (G.Z.); wangzheng@henu.edu.cn (Z.W.); 2 School of Computer and Information Engineering, Henan University, Kaifeng 475004, China; yangwei@henu.edu.cn; 3 School of Physics and Electronics, Henan University, Kaifeng 475004, China

**Keywords:** unsupervised feature selection, gene data, tumor classification, structure learning

## Abstract

Identifying the key genes related to tumors from gene expression data with a large number of features is important for the accurate classification of tumors and to make special treatment decisions. In recent years, unsupervised feature selection algorithms have attracted considerable attention in the field of gene selection as they can find the most discriminating subsets of genes, namely the potential information in biological data. Recent research also shows that maintaining the important structure of data is necessary for gene selection. However, most current feature selection methods merely capture the local structure of the original data while ignoring the importance of the global structure of the original data. We believe that the global structure and local structure of the original data are equally important, and so the selected genes should maintain the essential structure of the original data as far as possible. In this paper, we propose a new, adaptive, unsupervised feature selection scheme which not only reconstructs high-dimensional data into a low-dimensional space with the constraint of feature distance invariance but also employs $\ell_{2,1}$-norm to enable a matrix with the ability to perform gene selection embedding into the local manifold structure-learning framework. Moreover, an effective algorithm is developed to solve the optimization problem based on the proposed scheme. Comparative experiments with some classical schemes on real tumor datasets demonstrate the effectiveness of the proposed method.

## 1. Introduction

Cancers are now responsible for the majority of global deaths and are expected to rank as the leading cause of death. Thus, cancer may be the most important barrier to increasing life expectancy in every country in the world in the 21st century [1]. In the treatment of cancers, the correct diagnosis of the type and nature of tumors at as early a stage as possible is conducive to increased efficacy [2]. The development of DNA microarray technology has made it possible to study the causes of cancers from the level of genes, which greatly improves the accuracy of diagnosis and the curative effect related to cancer. Although DNA microarray data are usually high-dimensional, with the number of genes in a sample often running into thousands or even tens of thousands, there are often only a few key genes that determine specific tumors [3]. Since the original data contain excessive redundant genes and noise, directly using these data may lead to serious misclassification. Moreover, high-dimensional data also lead to a series of challenges such as a high storage cost and huge computation burden [4]. Therefore, selecting the important genes related to cancer classification from the original huge number of genes is one of the key research areas with respect to gene data classification.

Currently, many effective methods of gene selection have been proposed. These methods can be roughly divided into three categories—i.e., filter, wrapper, and embedded—depending on their evaluation manner [5]. The filter method employs the “certainty” metric to assign a score that reflects the ability of a gene to maintain the internal structure of data to determine the relevance between genes and specific cancers. However, as it neglects correlations among genes, this method may lose the important structural information underlying the original data. The wrapper method wraps genes into subsets and uses learning algorithms or predictive models to evaluate the importance of these subsets. However, the large number of subsets may induce a huge computational burden. The embedded method utilizes specific learning algorithm searching in the gene space for gene selection. In contrast with the other approaches, the embedded algorithm does not need to evaluate the classification ability of genes but only needs to select genes according to certain rules, leading to a lighter computational burden than the wrapped algorithm.

Embedded algorithms are divided into supervised feature (gene) selection (SFS) [6,7] and unsupervised feature selection (UFS) [8,9] approaches depending on whether they use label information. Typical SFS algorithms include SPFS [10], LLFS [11], mRMR [12], L21RFS [13], DLSR-FS [14], feature selection through sparse guidance [15,16], and so on. Although the above methods use the sparsity of the graph structure or regression model to reduce the misclassification induced by noise, the expensive and laborious label cost limits the wide application of SFS approaches [17,18]. When attempting to discover characteristic patterns in data without labels, UFS is more challenging. Representative algorithms of the UFS type include SPEC [19], FSSL [20], JELSR [21], EVSC [22], Laplacian Score [23], and so on.

Most of the above algorithms attempt to select features by uncovering the local manifold structure of data. More specifically, the above algorithms try to determine the embedding mapping which may reveal the low-dimensional manifold structure underlying the high-dimensional original gene data. Thus, the dimensionality reduction of the original gene data may be realized, and the inherent pattern of the data can even be found [24]. Generally, the local manifold of the original data may be usually represented in the form of graphs such as a samples pair similarity graph [10], k-NN graph [23], local linear embedding [25], and so on. In addition, besides the local structure of the original gene data, the global structure and the discriminant structure of the original gene data may also be explored to classify cancer [26,27,28]. However, these methods merely focus on presenting the local structure, while they ignore the maintenance of the global structure of the original gene data [29]; thus, their performance may be deteriorated by noise in the original data space.

Another challenge related to gene data classification is the dimension reduction of the original data. Since the original gene data are high-dimensional and have a complex topological structure, localizing the key genes related to cancer classification in the huge amount of original gene data is also challenging. Nie, Xu et al. [30] proposed a unified UFS framework of dimensionality reduction, which uses a minimization regression residual criterion to linearize project data into a low-dimensional subspace. However, similar to the above-mentioned methods, maintaining the global structure of the original gene data is not included in their work. Inspired by Nie’s work, in this paper, we propose a unified UFS framework with characteristics including gene selection, global and local structure learning from original gene data. In the proposed UFS framework, we design a regression function composed of three parts which satisfies the requirement of embedding mapping including dimensionality reduction and the maintenance of the global and local structures of the original gene data. Specifically, the multi-dimensional scaling (MDS) method is first used to project the original gene data from the high-dimensional space into a low-dimensional space on the constraint of the Euclidean distance invariant. Then, the sparse regression method is employed based on the minimized regression residual criterion to learn the reconstruction coefficient in the low-dimensional space, meaning that the global structure of the original data can be maintained in the course of the dimensionality reduction of the original data. Finally, a probabilistic neighborhood graph model based on sample genes is used to maintain the local manifold structure of the data. The contributions of this article are summarized as follows.

1. We combine structure learning and feature selection to propose a new feature selection framework. Since the MDS method is employed in the proposed framework to preserve the original space structure, which is reconstructed in a low-dimensional space, the proposed framework can preserve both the global structure and local structure underlying the original gene data;
2. The alternating direction method of multipliers (ADMM) is proposed to handle non-convex optimization related to the proposed framework. In addition, an efficient strategy related to the inverse of the high-dimensional matrix is also included in the proposed method;
3. The convergence and computational complexity of the proposed algorithm is discussed. Extensive experiments on multiple gene data demonstrate the superiority of our framework and method.

The rest of the paper is organized as follows. Section 2 briefly recalls the existing unsupervised embedded feature selection algorithms and introduces the MDS algorithm. Section 3 introduces the proposed approach and the optimization process. In Section 4, we analyze the convergence and parameter selection of the proposed algorithm. In Section 5, we conduct extensive experiments on multiple datasets and discuss and analyze several experimental results. In the last section, we present the conclusion and future prospects.

## 2. Related Work

In this section, we review several typical UFS algorithms.

In the past few years, UFS based on the spectral analysis technique has shown outstanding performance. Zhao and Liu [19] proposed the spectrum feature selection (SPEC) algorithm, which employs spectral analysis based on graph theory to select features with correlation. Due to the lack of an embedded learning process and the low sparsity of the graph caused by excessive samples, SPEC may be susceptible to noise and irrelevant features. Li, Yang et al. [28] proposed a non-negative discriminant feature selection algorithm (NDFS), which uses the correlation between discriminant information and features to select features. Specifically, NDFS first uses the spectral clustering technique to detect the structure underlying the original gene data and then learns the clustering label to construct the feature selection matrix, finally selecting features with discriminant information. Although the influence on the graph structure of noise is reduced by the structure learning and graph sparsity, NDFS can only work in the situation in which a linear relationship between the features and the clustering pseudo tags exists; moreover, the clustering tag technique employed by NDFS cannot fully capture the local structure information underlying the original data.

As mentioned above, the graph of the original gene data is susceptible to noise and irrelevant features; thus, it is necessary to reveal the data relationship in the low-dimensional subspace of the original gene data. Hou, Nie et al. [21] proposed the joint low-dimensional embedded learning and sparse regression (JELSR) feature selection method. However, their method merely focuses on low-dimensional manifold embedding, thus ignoring the maintenance of the global structure of original gene data, leading to some globally important information being missing. Ye and Zhang et al. [18] incorporated linear discriminant analysis (LDA), an adaptive structure based on spectral analysis and $\ell_{2,1}$-norm sparse regression into the joint learning framework of UFS. Although, their method employs the $\ell_{2,1}$-norm to enforce the row sparsity of the feature selection matrix, leading the projection matrix based on the LDA method to have the capability of feature selection, limitations of the traditional LDA method, such as suboptimal solutions and ignoring local manifolds, are also inherited. In this paper, we employ the multi-dimensional scaling (MDS) algorithm to reduce the dimensions of the original gene data and to maintain the global structure of the original gene data. In contrast to LDA and principal component analysis (PCA), the goal of MDS is not to preserve the maximum divisibility of the original data but to pay more attention to maintaining the internal characteristics of features underlying high-dimensional data.

## 3. Method

### 3.1. Nations

Gene expression data can be described as $X=[x_{1},x_{2},\cdots ,x_{n}]\in R^{d\times n}$, where $x_{i}\in R^{d}$ is the *i*-th sample, and *n* is the number of samples. Denote $L=\left[l_{1},l_{2},\cdots ,l_{n}\right]\in R^{n}$ as the true label vector, where $l_{i}\in \left\{1,\cdots ,C\right\}$ represents category of the *i*-th sample, *C* is the class number of the sample set. $I_{n}\in R^{n\times n}$ is an identity matrix. $1_{n}\in R^{n}$ is a vector with all elements equal to 1. Define the nonlinear operator ${\left(•\right)}_{+}=\max\left(•,0\right)$. The trace of $A=\left(a_{ij}\right)\in R^{n\times n}$ is written as Tr(A), and the $\ell_{2,1}$-norm of matrix A is defined as

(1) $$\left||A\right|{|}_{2,1}=\overset{d}{\underset{i=1}{\sum }}{\left(\overset{n}{\underset{j=1}{\sum }}a_{ij}^{2}\right)}^{\frac{1}{2}}$$

### 3.2. Proposed Objection Function

Inspired by the adaptive structure [29], we combine global structure learning and local manifold learning into the unified framework in order to uncover the important information underlying the original data; thus, the objective function corresponding to the proposed method can be formulated as

(2) minW,0≤pij≤1∑i=1npij=1||WTX−Y||F2+α||W||2,1+β∑i,jn(||WTxi−WTxj||2pij+λpij2)

where α and β are regularization parameters used to balance the adaptive structure learning and the feature selection coefficient matrix, and is the regularization parameter used to add a prior uniform distribution and to avoid a trivial solution. Y is the low-dimensional representation of the original dataset X. $P=\left(p_{ij}\right)\in R^{n\times n}$ is the neighborhood probability matrix, where $p_{ij}$ represents the probability that $x_{i}$ is connected with $x_{j}$, $0⩽p_{ij}⩽1$. Obviously, the probability of all samples being connected to $x_{i}$ should be satisfied following $\sum_{j=1}^{n}p_{ij}=1$.

**Figure 1:**
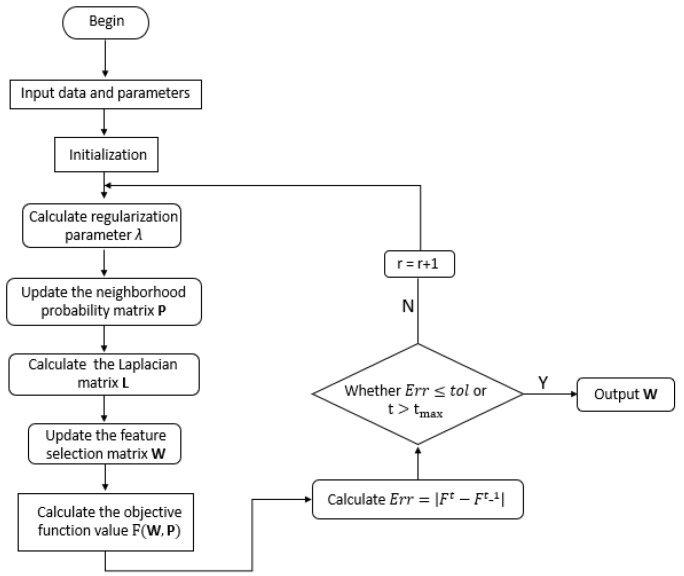
Block diagram of MDS-AUFS.

It can be found that the first two terms of the objective function utilize the minimum residuals criterion to learn the reconstruction coefficient of the original data in the low-dimensional space. As is known, the random mapping of the original data into the low-dimensional space may change the distances within the original data, leading the globe structure contained in the original data to twist. To map the original data into a low-dimensional space while maintaining its global structure, we employ the MDS method to transform X into Y since MDS has the ability to keep the sample distance of the original space the same as the sample distance of the transformed low-dimensional space. In the second term, the $\ell_{2,1}$-norm is used to force the row of W to be sparse, since the *i*-th row of the W matrix is related to the information of the *i*-th gene; this penalty term can enable the matrix W to perform feature selection. The third term of the objective function is the penalty term with a probability neighborhood matrix, which is employed to maintain the local structure of the gene-space manifold by using the prior information and to relieve the influence of uncorrelated genes on the local structure of manifolds.The block diagram of this work is shown in Figure 1.

### 3.3. Optimized

As it is composed of two constraint regularizations which contain two coupled, optimized variables, it may be difficult to derive the closed solution of the optimization problem described by Equation (2) directly. Inspired by the optimization methods in [31,32], we used an alternative iterative method which fixed one variable to update another variable to transform the optimization problem into multiple subproblems.

#### 3.3.1. Update P by Fixing W

When W is fixed, updating $P\;=\;[p_{1}^{T},p_{2}^{T},\cdots ,p_{n}^{T}{]}^{T}\in R^{n\times n}$ is equivalent to the following problem:

(3) $$\begin{array}{cc} \min_{\begin{array}{c} 0⩽p_{ij}⩽1\\ \sum_{j=1}^{n}p_{ij}=1 \end{array}}\; & \sum_{i.j}{∥W^{T}x_{i}-W^{T}x_{j}∥}_{2}^{2}p_{ij}+\lambda p_{ij}^{2} \end{array}$$

Let $b_{ij}=\frac{1}{2\lambda }\left|\right|W^{T}x_{i}-W^{T}x_{j}|{|}^{2}$, define matrix $B\;=\;{\left[b_{1}^{T},b_{2}^{T},\cdots ,b_{n}^{T}\right]}^{T}\in R^{n\times n}$. Problem (3) is equivalent to

(4) $$\begin{array}{cc} \min_{\begin{array}{c} 0⩽p_{ij}⩽1\\ 1_{n}p_{i}=1 \end{array}}\; & \frac{1}{2}\left|\right|p_{i}+b_{i}|{|}^{2}\;\forall i\in \left\{1,2,\cdots ,n\right\} \end{array}$$

The Lagrangian function of problem (4) is

(5) $$\Gamma \left(p_{i},\mu ,\nu_{i}\right)=\frac{1}{2}\left|\right|p_{i}+b_{i}|{|}^{2}-\mu \left(1_{n}p_{i}-1\right)-\nu_{i}^{T}p_{i}$$

where μ and $\nu_{i}$ are Lagrangian multipliers. According to the KKT condition [33], the optimal solution of problem (5) is

(6) $$p_{ij}={\left(-b_{ij}+\mu \right)}_{+}$$

By sorting each row of B into $\bar{B}$ in descending order [29], the following inequality holds:

(7) $$\left\{\begin{array}{c} {\bar{B}}_{ik^{\prime }}+\mu >0,\begin{array}{cc} for & k^{\prime }=1,\ldots ,k \end{array}\\ {\bar{B}}_{ik^{\prime }}+\mu <0,\begin{array}{cc} for & k^{\prime }=k+1,\ldots ,n \end{array} \end{array}\right.$$

Considering the probability constraint on $p_{i}$, we further get

(8) $$\mu =\frac{1}{k}(1-\overset{k}{\underset{d^{\prime }=1}{\sum }}{\bar{b}}_{id^{\prime }}{)}_{+}$$

Substituting Equation (8) into Equation (6), we obtain the optimal P:

(9) $$p_{ij}={\left(\sum b_{ij}-\frac{1}{k}\left(1-\overset{k}{\underset{d^{\prime }=1}{\sum }}{\bar{b}}_{id^{\prime }}\right)\right)}_{+}$$

Similar to the method in [29], we set the regularization parameters λ according to *k*, which is the number of neighbors.

(10) $$\lambda =\frac{1}{2n}\overset{n}{\underset{i=1}{\sum }}\left(k{\bar{b}}_{i,k+1}-\overset{k}{\underset{j=1}{\sum }}{\bar{b}}_{ij}\right)$$

#### 3.3.2. Update W by Fixing **P**

Once P is fixed, updating W in (2) is equivalent to the following problem:

(11) $$\begin{array}{cc} \min_{W} & \left|\right|W^{T}X-{Y||}_{F}^{2}+\alpha ||W|{|}_{2,1}+\beta \sum_{i,j}^{n}\left(||W^{T}x_{i}-W^{T}x_{j}|{|}^{2}p_{ij}\right) \end{array}$$

Let $L_{p}=D_{p}-\frac{\left(P+P^{T}\right)}{2}$, where $D_{p}$ is a degree matrix with the *i*-th principal diagonal element being $\sum_{j}{(p}_{ij}+p_{ji})/2$. The optimization problem in (11) for updating W is equivalent to the following problem:

(12) $$\begin{array}{cc} \min_{W} & \left|\right|W^{T}X-{Y||}_{F}^{2}+\alpha ||W|{|}_{2,1}+2\beta Tr\left(W^{T}\mathrm{XL}X^{T}W\right) \end{array}$$

Although the optimization problem is complex, the regularization term $\alpha ||W|{|}_{2,1}$ is not differentiable. To handle this problem, denote $M\in R^{d\times d}$ as a diagonal matrix with the *i*-th diagonal element being $m_{ii}=\frac{1}{2\sqrt{\left|\right|w_{i}{\left|\right|}_{2}^{2}+\varepsilon }}$, where ε is a small value. The problem (12) can be rewritten as

(13) $$\begin{array}{cc} \min_{W} & \left|\right|W^{T}X-Y{\left|\right|}_{F}^{2}+\alpha Tr\left(W^{T}\mathrm{MW}\right)+2\beta Tr\left(W^{T}\mathrm{XL}X^{T}W\right) \end{array}$$

Thus, the analytical solution of problem (13) is

(14) $$W={\left(X\left(I_{n}+\beta L\right)X^{T}+\alpha M\right)}^{-1}XY^{T}$$

It can be found that solution of Equation (14) involves the inverse of the d×d matrix. Since the gene dimensionality *d* is much larger than the number of samples *n* in the gene expression data, the inverse operation of a large matrix can considerably increase the computational overhead of the proposed algorithm. Similar to the methods in [17], we can convert a d×d matrix inverse problem into an n×n one, as shown in (15):

(15) $$W=\mathrm{VX}{\left(\left(I_{n}+\beta L\right)X^{T}\mathrm{VX}+I_{n}\right)}^{-1}Y^{T}$$

where $V=\frac{1}{\alpha }M^{-1}$. The procedure of the proposed algorithm is summarized in Algorithm 1.
**Algorithm 1.** AUFS-MDS**Input:**  Gene expression data matrix $X\in R^{d\times n}$;  Number of nearest neighbors *k*; Number of real label *C*;  Low dimensional representation *q*;  Regularization parameter α and β;  Number of selected genes *s*;**Output:**  The top *s* ranked features as the results of feature selection.**1:**
To generate a low dimensional representation $Y\in R^{q\times n}$ of X by MDS;**2:**
Initialize $W\in R^{d\times q}$ as a random matrix; $B\in R^{n\times n}$ by setting $b_{ij}=\left|\right|x_{i}-x_{j}|{|}^{2}$;**Repeat****3:**
Calculate λ based on (10);**4:**
Calculate P based on (9);**5:**
Calculate $L=D-(P+P^{T})/2$;**6:**
Calculate W by solving the problem (15);**7:**
Update M with the *i*-th diagonal element as $m_{ii}=\frac{1}{2\sqrt{\left|\right|w_{i}{\left|\right|}_{2}^{2}+\varepsilon }}$;**8:until** Convergence;Sort all genes based on $\left|\right|w_{i}|{|}_{2}$ in descending order. The top *s* ranked genes are selected.

## 4. Analysis

### 4.1. Convergence Analysis

We introduce a lemma [13] for discussing the convergence with the variable W of the proposed AUFS-MDS algorithm.

**Lemma** **1.** *For any nonzero vectors $a,b\in R^{m}$, the following result is obtained:*

(16) $$\begin{array}{c} \left||a\right|{|}_{2}-\frac{{\left||a|\right|}_{2}^{2}}{2{∥b∥}_{2}}⩽{∥b∥}_{2}-\frac{{∥b∥}_{2}^{2}}{2{∥b∥}_{2}} \end{array}$$

**Theorem** **1.** *The objective function value of the AUFS-MDS algorithm can be monotonically reduced to convergence by updating the variable W.*

**Proof** **Theorem** **1.** Problem (2) can be written as

(17) $$\begin{array}{cc} F(W)= & \left|\right|W^{T}X-{Y||}_{F}^{2}+\alpha ||W|{|}_{2,1}+\beta \sum_{i,j}^{n}\left(||W^{T}x_{i}-W^{T}x_{j}|{|}^{2}p_{ij}+\lambda p_{ij}^{2}\right) \end{array}$$

Let $L\left(W\right)=\left|\right|W^{T}X-Y{\left|\right|}_{F}^{2}$, $J\left(W\right)=\sum_{ij}^{n}\left(||W^{T}x_{i}-W^{T}x_{j}|{|}^{2}p_{ij}+\lambda p_{ij}^{2}\right)$, Equation (17) is equivalent to following:

(18) $$F\left(W\right)=L\left(W\right)+\beta J\left(W\right)+\alpha ||W|{|}_{2,1}$$

According to the AUFS-MDS algorithm, the following inequality holds when W is updated:

(19) $$F\left(W^{t+1}\right)⩽F\left(W^{t}\right)$$

Known ${∥W∥}_{2,1}=\sum_{i=1}^{d}(\sum_{j=1}^{q}w_{ij}^{2}{)}^{1/2}$, let ${∥{\bar{w}}_{i}∥}_{2}={\left(\sum_{j=1}^{q}w_{ij}^{2}\right)}^{1/2}$, the inequality related to Equation (18) is as follows:

(20) $$L\left(W^{t+1}\right)+J\left(W^{t+1}\right)+\alpha {∥\left(W^{t+1}\right)∥}_{2,1}+\alpha \overset{d}{\underset{i=1}{\sum }}\left(\frac{{∥{\bar{w}}_{i}^{t+1}∥}_{2}^{2}}{2∥{\bar{w}}_{i}^{t}∥}-{∥{\bar{w}}_{i}^{t+1}∥}_{2}\right)\;⩽L\left(W^{t}\right)+J\left(W^{t}\right)+\alpha {∥\left(W^{t}\right)∥}_{2,1}+\alpha \overset{d}{\underset{i=1}{\sum }}\left({\frac{{∥{\bar{w}}_{i}^{t}∥}_{2}^{2}}{2∥{\bar{w}}_{i}^{t}∥}}_{2}-{∥{\bar{w}}_{i}^{t}∥}_{2}\right)$$

According to Lemma 1, we obtain

(21) $$\frac{{∥{\bar{w}}_{i}^{t+1}∥}_{2}^{2}}{2{∥{\bar{w}}_{i}^{t}∥}_{2}}-{∥{\bar{w}}_{i}^{t+1}∥}_{2}⩾\frac{{∥{\bar{w}}_{i}^{t}∥}_{2}^{2}}{2{∥{\bar{w}}_{i}^{t}∥}_{2}}-{∥{\bar{w}}_{i}^{t}∥}_{2}$$

Combining (20) and (21), we get the following result:

(22) $$L\left(W^{t+1}\right)+J\left(W^{t+1}\right)+\alpha {∥\left(W^{t+1}\right)∥}_{2,1}⩽L\left(W^{t}\right)+J\left(W^{t}\right)+\alpha {∥\left(W^{t}\right)∥}_{2,1}$$

Inequality (22) indicates that the objective function in problem (2) will decrease monotonically with each iteration. □

In addition, as we have presented in Section 2, the objective function in problem (2) is convex with respect to variable W; thus the above iteration will lead to convergence because the objective function has a lower bound. Although we have shown the convergence with the variable of the objective function in problem (2), the convergence of W itself is still unknown. To show the convergence of W, the variance of W changing with iterations, which is described in (23), is discussed in the next section.

(23) $$Err\left(W\right)=\overset{d}{\underset{i=1}{\sum }}\left|{∥{\bar{w}}_{i}^{t+1}∥}_{2}-{∥{\bar{w}}_{i}^{t}∥}_{2}\right|$$

### 4.2. Parameter Determination

As is known, the determination of parameters related to regular terms is still an open problem. In proposed framework, the first parameter *q* denotes the low-dimensional embedded dimension of the original high-dimensional sample X, which is referred to as the intrinsic dimension in manifold learning. In [34], two strategies were proposed to select the value of *q* based on the uncertainty of entropy. In this paper, to facilitate the experiment and without losing generality, *q* is set to be equal to the number of sample classes in the experiment. The second parameter is *s*, which denotes the number of genes selected. We vary *s* within a certain range as it is difficult to determine without prior knowledge. Finally, the regularization parameters α and β are determined by a grid search according to experience.

## 5. Experiment

In this section, we present extensive experiments that were conducted to evaluate the performance of our proposed unsupervised gene selection algorithm.

### 5.1. Datasets

The experiments were conducted on five publicly available cancer gene datasets, including a lung dataset, a colon dataset, a lymphoma dataset, a glioma dataset and a leukemia dataset. All data were downloaded from https://jundongl.github.io/scikit-feature/datasets.html (accessed on 1 May 2021), and details of the data are summarized in Table 1.

### 5.2. Contrast Algorithm

To evaluate the effectiveness of the proposed MDS-AUFS algorithm, we compared it with six classical unsupervised feature selection algorithms, the details of which are described as follows.

- URAFS [9] embeds the local geometric structure of data into the manifold learning framework by introducing the graph regularization term based on the principle of maximum entropy into the GURM model, leading to the irrelevant features of the original data being filtered out;
- UDFS [25] embeds discriminative analysis and the $\ell_{2,1}$-norm into the feature selection framework to select discriminative features and informative features;
- SPEC [19] is a unified feature selection framework based on graph theory and is used to select relevant features by combining supervised feature selection and unsupervised feature selection;
- NDFS [28] utilizes the discriminant information and correlation of features to select feature subsets. Specifically, the method combines cluster labels learned by the spectrum clustering algorithm with the feature selection matrix to finally select the most discriminant features;
- LLCFS [35] integrates local structure learning and feature selection into a unified framework. Specifically, LLCFS embeds weighted features into the regularization term of the local clustering learning algorithm and selects features according to their weight;
- JELSR [27] is based on an unsupervised learning structure and combines embedding learning with sparse regression to select features.

### 5.3. Experimental Settings

#### 5.3.1. Parameter Settings

There are some parameters that needed to be set in advance. We set k=5 for all the datasets to specify the size of neighborhoods and make the low-dimensional *q* equal to the number of real classes *C*. For all datasets, the number of genes selected *s* was set as 5, 10, 15, 20, 25, 30, 35, 40, 45 and 50, respectively. Regularization parameters related to sparse terms and structure learning are denoted by α and β, respectively, and their values were set as shown in Table 2 according to different datasets.

#### 5.3.2. Evaluation Metrics

We employed the k-means clustering algorithm to evaluate the accuracy (ACC) of the proposed method which is described in (24) [36].

(24) $$A\mathrm{cc}=1/n\sum_{i=1}^{n}\delta (map\left(c_{i}\right),I_{i})$$

where $c_{i}$ represents the cluster label of $x_{i}$, and $I_{i}$ represents the real label of $x_{i}$. δ(•) is the δ-function. map(•) represents an optimal mapping function, which projects each cluster label into the real label by using the Kuhn–Munkres algorithm [37]. Apparently, a larger ACC shows better clustering performance.

### 5.4. Experiment and Discussion

Four group comparison experiments were implemented to demonstrate the performance of the proposed algorithm including its clustering ability, convergence, computation complexity and sensitivity with regularization parameters. The first group experiment compared the clustering ability of the proposed algorithm with other algorithms in terms of the selected number of genes. The second group experiment showed the convergence of the proposed algorithm. The third group experiment analyzed the computational complexity of the proposed algorithm in terms of the number of samples and the number of genes. The last group experiment showed the impact of the regularization parameters on the performance of the proposed algorithm.

#### 5.4.1. ACC Evaluation Index

**Figure 2:**
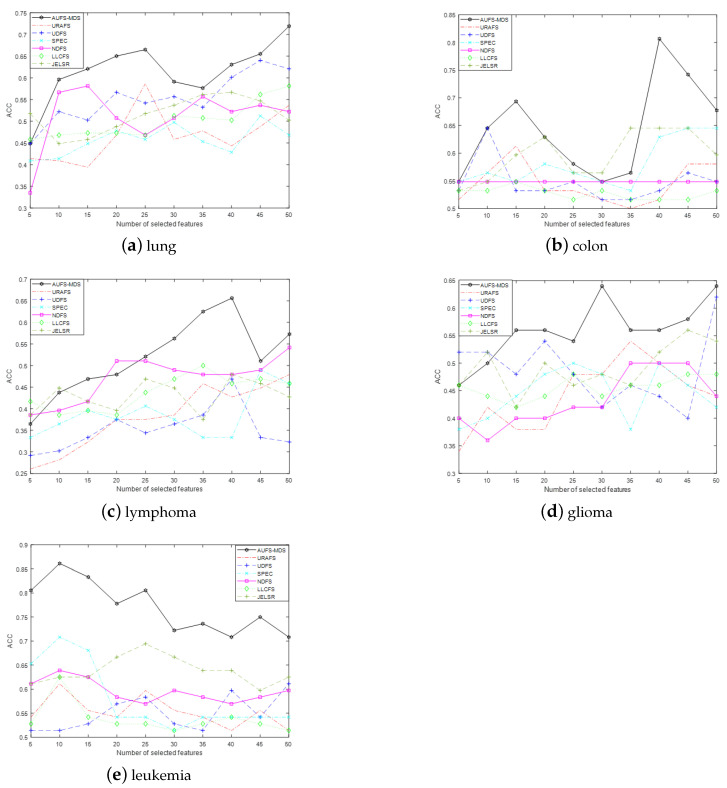
Clustering accuracy of all the methods on five different datasets.

**Figure 3:**
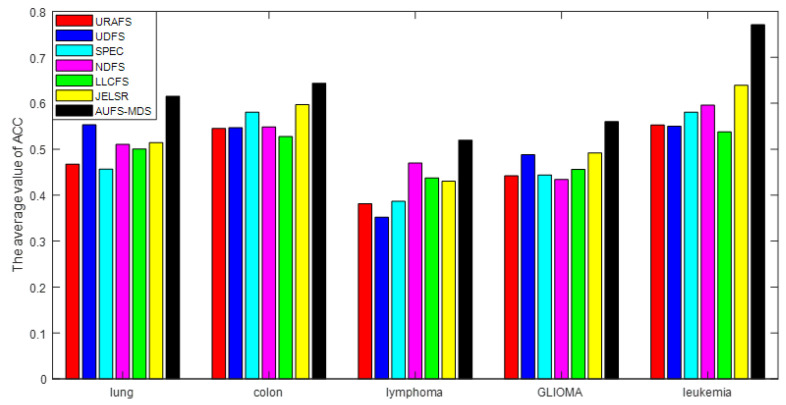
The average ACC of all the methods for five different datasets.

We evaluated the performance of our approach regarding feature selection using comparison experiments with several typical feature selection methods: URAFS, UDFS, SPEC, NDFS, LLCFS and JELSR. As we employed the k-means method, which is sensitive to the initialization parameters, to cluster the original data, to reduce the impact of the initialization parameters on the performance of the k-means method, we repeated the clustering 20 times with random initialization parameters and then plot the ACC with changing numbers of selected genes [38]. The optimal results and average results in the 20 experiments are described in Figure 2 and Figure 3, respectively.

To further demonstrate the performance of the proposed algorithm, in Table 3, we compare the maximum ACC of MDS-AUFS with that of the other algorithms on five different cancer gene datasets. In this table, the best results are written in bold, and the second-best results are underlined.

From Figure 2a–d, ACC indicators of other types of cancer except leukemia show an overall increasing trend in the initial stage of gene selection, while this begins to decline as the number of selected genes further increases. In addition, MDS-AUFS always achieves the maximum clustering ACC with fewer genes. It is may be inferred that all algorithms may achieve good performance when more keys gene are selected, and the proposed algorithm shows the best performance of all of the approaches; moreover, once all keys gene have been selected, irrelevant or redundant genes can be introduced into the algorithm as the number of selected genes further increases, leading to the performance of algorithms declining. From Figure 2e, we can see that the ACC of almost all algorithms show a decreasing trend, which implies that there is a small number of key genes related to leukemia. Furthermore, the maximum number of selected genes with respect to ACC in all methods is different in different gene datasets. For example, the number of selected genes is 40 when MDS-AUFS achieves optimal performance on colon data, while it is 10 for leukemia data. We have reason to believe that the number of key genes in different types of cancer is also different. Obviously, accurately selecting the key genes or gene subsets that contain the most key genes is important in cancer classification.

Figure 3 shows the average ACC of all algorithms in five datasets. It is easy to see that the ACC of the MDS-AUFS algorithm is significantly higher than that of other algorithms on different cancer gene datasets, which indicates that MDS-AUFS has the best robustness. Table 3 shows the optimal performance of all algorithms. As is evident, MDS-AUFS always achieves the best evaluation performance.

#### 5.4.2. Convergence of MDS-AUFS

**Figure 4:**
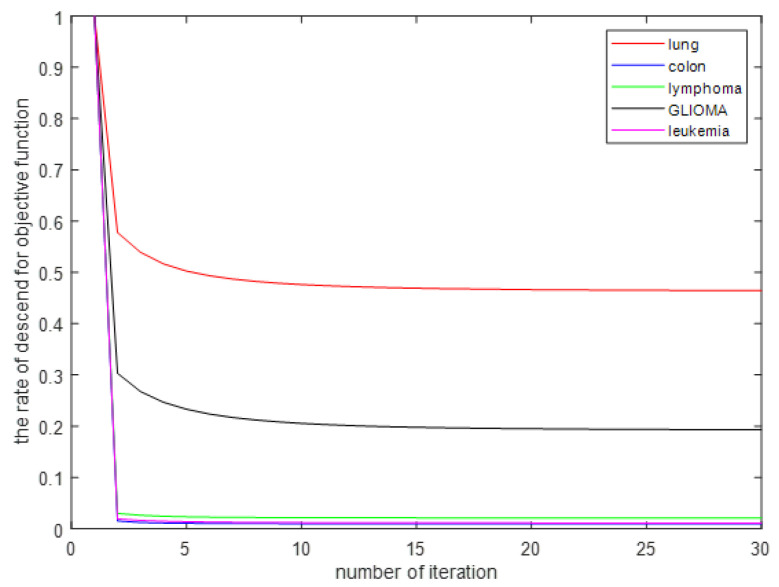
The convergence of MDS-AUFS on five gene datasets.

In this section, some experiments are presented that show the convergence of the iterative process of the MDS-AUFS algorithm. To intuitively observe the overall convergence of the MDS-AUFS algorithm, we normalized the value of the objective function after each iteration. As shown Figure 4 MDS-AUFS shows good convergence and quickly converged in all gene datasets, and it could achieve convergence in general in about 10 iterations.

**Figure 5:**
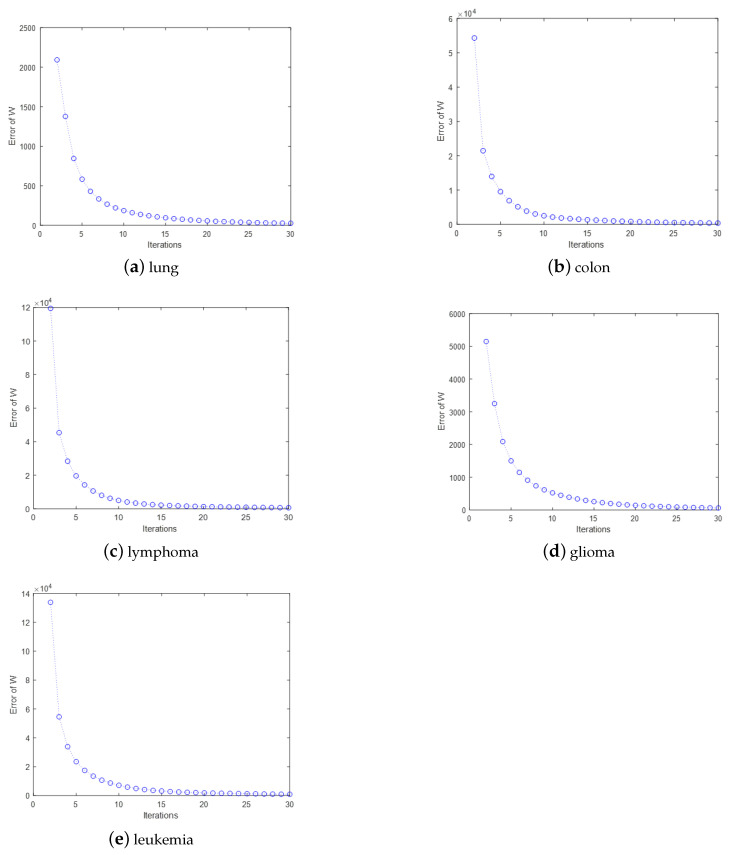
The convergence of W on five gene datasets.

At the same time, we also show the convergence process of the gene selection matrix on different gene datasets, as shown in Figure 5.

#### 5.4.3. Computational Complexity Analysis

In this section, we analysis the computational complexity and the running time of the proposed method and then compare it with several compared algorithms. The procedure of the proposed MDS-AUFS method is summarized in Algorithm 1. The time complexity of computing the low-dimensional representation of Y by MDS is $O\left(n^{2}q\right)$. It will stop when the objective function of problem (2) tends to a constant or the change is very close to zero. The most time-consuming operation of Algorithm 1 is solving problem (17) in the sixth step. We can convert a d×d matrix inverse problem to an n×n problem; in doing so, the time complexity of Algorithm 1 at each iteration becomes $O\left(\min{\left\{n,d\right\}}^{3}\right)$. Table 4 exhibits the complexity of all methods.

We also selected two representative datasets, lung and leukemia, for the purpose of demonstrating the influence of the sample number and dimension on the complexity of MDS-AUFS. As can be seen from Table 1, the lung dataset had the largest number of samples of all datasets, while samples in the leukemia dataset had the largest number of genes. We considered the algorithms’ running time when the number of genes was 10, 30 and 50, respectively. Our calculation was performed using MATLAB2019a on a 3.2 GHz Windows computer. Table 5 and Table 6 list all algorithms’ computing time when choosing a number of different genes. We can obtain the following conclusion according to the analysis:

- MDS-AUFS runs faster on all datasets. It is fast because the local structure of SPEC does not involve a learning process;
- We only consider the computational complexity theoretically. The time consumption may be different in real applications because we have not considered the influence of iteration in the above analysis;
- The calculation costs of different methods are determined by different factors. For example, MDS-AUFS runs in a short time for each iteration and has a significant speed advantage over other methods when *d* is large. The approach benefits from the conversion of the high-dimensional matrix inverse into the low-dimensional matrix inverse, which was designed in the optimization of MDS-AUFS;
- The computational complexity of all algorithms is not related to the selection of *s*.

#### 5.4.4. Sensitivity of Regularization Parameters α and β

**Figure 6:**
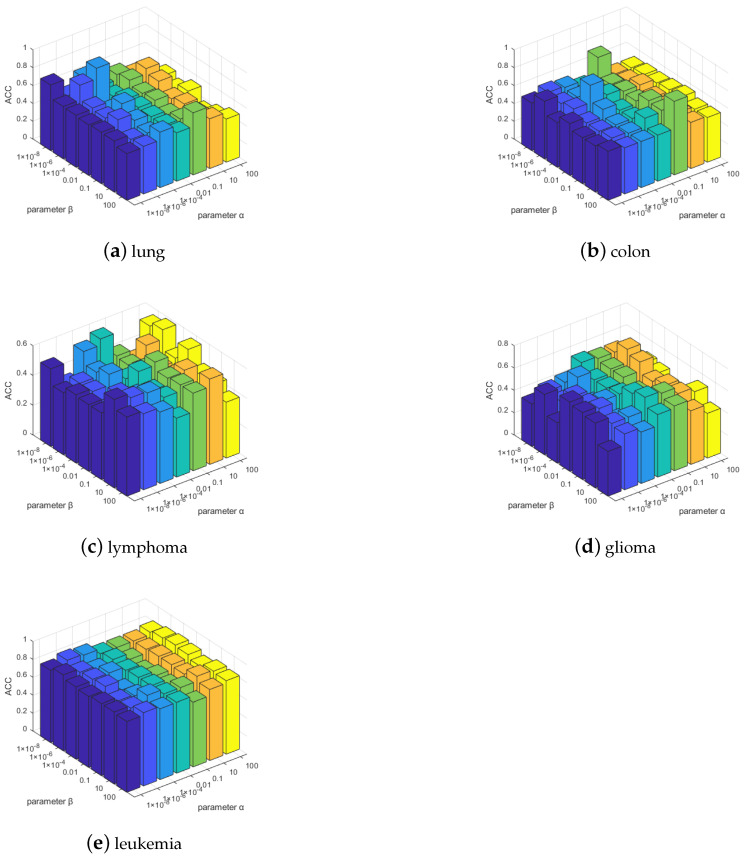
ACC of different parameter combinations on all gene datasets.

We show the ACC of the MDS-AUFS algorithm under different parameter combinations. Because the question of the determination of parameters is still open, we obtained α and β from $\left\{10^{-8},10^{-6},10^{-4},0.01,1,10,100\right\}$ by a grid search method according to experience. From Figure 6, parameters α and β in different combinations can be seen to lead to different performance levels for ACC using MDS-AUFS. To fairly compare different unsupervised feature selection algorithms, we used the grid search method to select the optimal combinations and demonstrated the ACC performance of these combinations.

## 6. Conclusions

In this paper, we present an adaptive, unsupervised feature algorithm that combines gene selection and structure learning into a unified framework of sparse representation. Specifically, the original high-dimensional data is first sparse-reconstructed into the low-dimensional space based on the MDS structure invariant constraint. Then, the probabilistic neighborhood relationship is introduced to learn the local manifold structure of the gene data. Moreover, the ADMM algorithm is employed to handle the above non-convex structure learning problem. The effectiveness of the proposed method is demonstrated by comparative experiments with some classical algorithms on five real cancer gene datasets. In future work, we will further explore the data structure information capturing method including key feature location and redundant feature detection. Another open problem is the parameter selection related to the MDS method; it is empirically determined in this paper and should be deeply discussed in future work.

## Figures and Tables

**Table 1:** Details of the datasets.

Datasets	Instances	Genes	Classes
Lung	203	3312	5
Colon	62	2000	2
Lymphoma	96	4026	9
Glioma	50	4434	4
Leukemia	72	7070	2

**Table 2:** The regularization parameters of datasets.

	α	β
Lung	0.1	$10^{-6}$
Colon	0.1	$10^{-8}$
Lymphoma	10	$10^{-6}$
Glioma	10	$10^{-6}$
Leukemia	0.1	$10^{-6}$

**Table 3:** Maximum clustering accuracy of different methods on five different datasets.

Methods	URAFS	UDFS	SPEC	NDFS	LLCFS	JELSR	MDS-AUFS
Lung	0.5862	0.6404	0.5123	0.5813	0.5813	0.5665	**0.7192**
Colon	0.6129	0.6452	0.6452	0.5484	0.5484	0.6452	**0.8065**
Lymphoma	0.4792	0.4688	0.4896	0.5417	0.5000	0.4479	**0.6563**
Glioma	0.5400	0.6200	0.5000	0.5000	0.4800	0.5600	**0.6400**
Leukemia	0.6111	0.6111	0.7083	0.6389	0.6250	0.6944	**0.8611**

**Table 4:** Comparison of the main computational complexity.

Methods	Computational Complexity
DUCFS	$O\left(d^{3}+n^{2}d\right)$
JELSR	$O\left(d^{3}+n^{2}q\right)$
LLCFS	$O\left(n^{3}\right)$
MCFS	$O\left(d^{3}+n^{2}q+nd^{2}\right)$
NDFS	$O\left(d^{3}+n^{2}c\right)$
SPEC	$O\left(n^{2}d\right)$
UDFS	$O\left(d^{3}+n^{2}c\right)$
URAFS	$O\left(d^{3}+n^{2}d+n^{2}\right)$
MDS-AUFS	$O\left(\min\left\{n,d^{3}\right\}+n^{2}q^{}\right)$

**Table 5:** The computation time of different methods on the lung dataset (unit: second).

*s*	MDS-AUFS	URAFS	UDFS	SPEC	NDFS	LLCFS	JELSR
10	21.48	47.97	116.96	0.13	94.20	4.55	16.35
30	21.34	49.51	116.43	0.12	94.14	4.25	16.02
50	21.25	48.36	116.54	0.11	93.26	4.35	16.01

**Table 6:** The computation time of different methods on lung and leukemia datasets (unit: second).

*s*	MDS-AUFS	URAFS	UDFS	SPEC	NDFS	LLCFS	JELSR
10	258.52	367.829	601.926	0.088	363.118	2.002	51.241
30	279.43	367.883	601.162	0.085	356.375	2.065	52.175
50	256.77	366.948	601.628	0.080	354.011	2.075	52.500

## Data Availability

The data presented in this study are available on request from the first author.
